# A Comparison of Colorimetric Assessment of Vaginal pH with Nugent Score for the Detection of Bacterial Vaginosis

**DOI:** 10.1155/2017/1040984

**Published:** 2017-02-15

**Authors:** Matthew K. Hoffman, Mrutyunjaya B. Bellad, Umesh S. Charantimath, Avinash Kavi, Jyoti M. Nagmoti, Mahantesh B. Nagmoti, Ashalata A. Mallapur, Geetanjali M. Katageri, Umesh Y. Ramadurg, Sheshidhar G. Bannale, Amit P. Revankar, M. S. Ganachari, Richard J. Derman, Shivaprasad S. Goudar

**Affiliations:** ^1^Department of Obstetrics & Gynecology, Christiana Care Health System, Newark, DE, USA; ^2^KLE University Jawaharlal Nehru Medical College, Belgaum, Karnataka, India; ^3^S. Nijalingappa Medical College and HSK Hospital and Research Centre, Bagalkot, Karnataka, India; ^4^Thomas Jefferson University, Philadelphia, PA, USA

## Abstract

*Background*. A Nugent score > 7 has been defined as the gold standard for the diagnosis for bacterial vaginosis (BV), though it is resource intensive and impractical as point of care testing. We sought to determine if colorimetric assessment of vaginal pH can accurately predict the occurrence of BV.* Methods*. We performed a planned subanalysis of 1,216 pregnant women between 13 0/7 and 19 6/7 weeks who underwent vaginal examination as part of a randomized controlled trial. Using a standardized technique, specimens were obtained for colorimetric assessment and two separate slides for Gram staining. These slides were subsequently evaluated by two independent blinded microbiologists for Nugent scoring.* Results*. Interrater reliability of the interpretation of the Nugent score was excellent (intraclass correlation-individual 0.93 (95 CI 0.92 to 0.94) and average 0.96 (95% CI 0.95 to 0.97)). The sensitivity of an elevated pH > 5 for a Nugent score > 7 was 21.9% while the specificity was 84.5%. The positive predictive value in our population was 33.7% with a negative predictive value of 75.0%.* Conclusion*. Though the Nugent score is internally accurate, the prediction of BV using vaginal pH alone has poor sensitivity and specificity.

## 1. Background

Bacterial vaginosis (BV) remains the most common form of vaginitis affecting women globally [1] and has been linked to several poor outcomes including preterm birth [2] and posthysterectomy infection [3]. Nonetheless, 29% of US women are noted to meet diagnostic criteria of BV, though the majority are clinically asymptomatic [4]. Recently, genetic microbiome studies have demonstrated that the occurrence of BV represents an absence of acid forming morphotypes of lactobacillus [5]. This lack of lactobacilli is frequently accompanied by an overgrowth of* Gardnerella vaginalis* forming an infected biofilm and creating a permissive environment for the overgrowth of numerous anaerobic Gram negative rods [6]. Nonetheless, the vaginal microbiome, as defined by Nugent scoring, is noted to vary dramatically by region of the world [7].

Several prior studies have suggested that vaginal pH alone may be an accurate marker for the detection of BV [8–10]. These high correlations have not been witnessed in other studies. It is thus important to establish the accuracy of vaginal pH to detect BV in an area where the resource intensive approaches to Gram stain and light microscopy may not be readily available. We thus sought to determine the ability of vaginal pH to detect BV as defined by the Nugent score amongst pregnant women in a low middle income setting in southern India.

## 2. Methods

Prior to the initiation of the study, Institutional Review Board approval was obtained from both participating institutions (Jawaharlal Nehru Medical College, Belgaum, India, and Christiana Care Health Services, Newark, Delaware). This trial was a planned substudy of a prospective individually randomized trial of pregnant women with an elevated vaginal pH (≥5.0) who would be treated with Clindamycin or placebo (ClinicalTrials.Gov NCT01800825, ICTR CTRI/2013/07/003799). Pregnant women between the gestational ages of 13 0/7 and 19 6/7 weeks were invited to participate in the trial. To be included women were to have a singleton gestation with no anomalies by history. Women were likewise excluded if they had a history of taking antibiotics in the last 14 days, had a history of vaginal bleeding in the last 3 days, had a symptomatic vaginal discharge, or were unable to consent (age < 18 years with no provisions for family/husband consent). Consistent with the guidelines of the ethics review committee, before each examination, a woman provided consent in her local language under the direct observation of trained field staff.

Our primary outcome was the presence of BV as defined as a Nugent score of 7 or greater [11]. Though competing definitions for BV exist, such as Amsel's criteria, the Nugent scoring system was chosen as it has been acknowledged as the gold standard [11, 12].

Prior to field initiation, certified Auxiliary Nurse Midwives (ANMs) were trained in a standardized methodology for both obtaining slides for Gram stain and vaginal pH using a video and direct observation by a central team member. Using a standardized speculum exam, specimens were taken from the lateral vaginal sidewalls. Gram stains were obtained using 2 separate acrylic swabs and then immediately plated on a clean glass slide that was allowed to air dry. These slides were transported to a central reading area where they underwent Gram stain. Nugent score was then read by two independent pathologists in accordance with accepted techniques [11]. Women who had a Nugent score of 7 or greater were deemed to have bacterial vaginosis. In cases wherein the two pathologists disagreed and with one scoring below 7, the higher score was chosen to define the presence or absence of bacterial vaginosis.

Vaginal pH was determined by directly placing a small portion of pH paper in the same location that the vaginal swab for the Gram stain was obtained from and it remained until being saturated with vaginal fluid. The pH was then evaluated after the pH paper had been allowed to dry after 60 seconds. PH paper was universally obtained from Micro-Essential Laboratories (Hydrion 345 S/R Dispenser; Brooklyn, NY) and is able to discriminate pH in 0.5 moles per liter increments between 3.0 and 5.5. These were recorded and read independently of the Gram stains.

Statistical analysis consisted of performing an intraclass correlation (two-way mixed effect model) between the two scoring microbiologists for both Nugent score and the occurrence of BV (Nugent score ≥ 7). Comparison of the presence of BV defined by an elevated vaginal pH (≥5) versus BV defined by Nugent score was likewise compared using a pairwise correlation. Summary statistics were performed using simple univariate modelling. All statistical analysis were performed using STATA 14.0 (College Station TX).

## 3. Results

**Figure 1:**
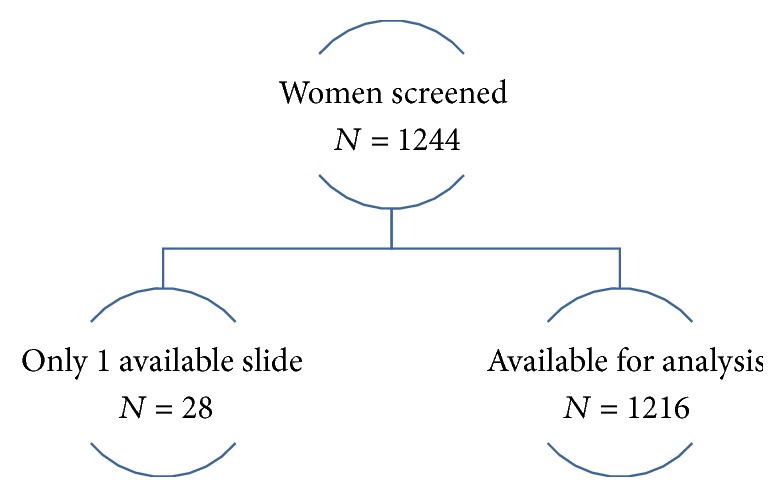


A total of 6,473 women underwent screening in the parent study; 26.7% (*N* = 1,728) were found to have a vaginal pH ≥ 5. Of this cohort, a total of 1,244 women underwent screening with both a vaginal Gram stain and a vaginal pH, of which 1,216 (97.6%) met the entrance requirement (see Figure 1). Twenty-eight women were eliminated due to the lack of a second slide. Demographics of the participants are presented in Table 1. Our participants can best be summated as a young healthy but underweight cohort. This cohort is consistent with participants of our prior studies and the general population [13].

As determined by a Nugent score of 7 or greater, 17.4% of our participants had bacterial vaginosis. With reference to the Nugent score, there was a very high correlation between the two pathologists both between each other and the mean value. The intraclass correlation between raters was 0.929 (95% CI 0.920 to 0.936) and between the average was 0.963 (95% CI 0.959 to 0.967) (*P* value < 0.001). This suggests that Nugent score is a highly accurate and consistent test.

**Figure 2:**
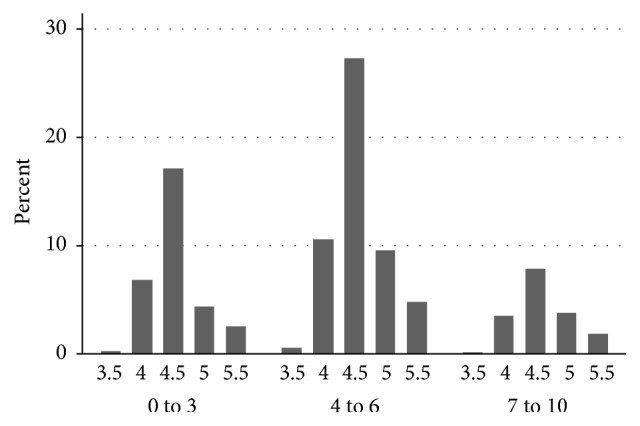
% distribution by pH and Nugent grouping.

In contrast, vaginal pH fared poorly as a predictor of bacterial vaginosis defined as a Nugent score of 7 or greater. This is graphically illustrated in Figure 2, wherein the distribution of vaginal pH values is shown by Nugent groups (0 to 3, 4 to 6, and 7 to 10). In this graph one can see that the frequency of vaginal pH values appears to be normally distributed, not skewed with the higher pH values in the 7 to 10 group. The pairwise correlation between a pH ≥ 5 and an elevated Nugent score was 0.66 (*P* value of 0.021). The sensitivity of an elevated pH ≥ 5 by Nugent score was 21.7% while the specificity was 84.1%. The positive predictive value in our population was 31.1% with a negative predictive value of 76.4%.

## 4. Discussion

The clinical diagnosis of bacterial vaginosis has long been made using Amsel's criteria (elevated vaginal pH, presence of clue cells, milky discharge, and positive whiff test) or Nugent's criteria [14]. Through microbiotics, we have come to understand that bacterial vaginosis is a complex entity characterized largely as the absence of acid forming lactobacillus and a concomitant proliferation of other anaerobic bacteria [5]. This lack of acidity allows the proliferation of other organisms (microbial dysbiosis) which may be causative of underlying disease [15]. As lactobacilli play a key role in acidifying the vagina, it is not surprising that vaginal pH remains part of Amsel's criteria in making the clinical diagnosis of bacterial vaginosis.

Though we were able to demonstrate that an elevated vaginal pH is significantly associated with bacterial vaginosis as defined by Nugent's criteria, our measurement of vaginal pH had poor sensitivity and specificity compared to other publications. Amongst our participants, only 21.7% of women with an elevated vaginal pH (defined as vaginal pH or greater than or equal to 5.0.) had bacterial vaginosis. This finding is much more consistent with other reports that have found vaginal pH to be a relatively poor predictor of BV [8, 10]. This may in part be explained by the fact that vaginal microbiome of southern India may be markedly different than that in other countries [7].

Though broadly accepted as the gold standard, it is important to remember that the Nugent score reflects the relative ratios of large Gram positive rods (lactobacillus), small Gram variable rods, and curved Gram variable rods [11]. Whether these findings are diagnostic of BV and are associated with both disease and poor obstetrical outcomes in rural India where the microbiome may be different is unclear [7]. Further complicating the issue, recent genomic studies have demonstrated that “healthy” women who lack appreciable colonies of lactobacilli are relatively common [16, 17]. These women appear to have other bacteria that are capable of acidifying the vagina, promoting microbial health. In such women, Nugent's criteria may not be an appropriate marker of the health of the vaginal microbiome.

Perhaps more important than how the diagnosis of bacterial vaginosis is derived is that an elevated vaginal pH represents an atypical vaginal microbiome free of acid forming organisms. Such an environment has been shown to provide permissive growth of pathological organisms like mycoplasma amongst others [18]. Several investigations have been able to link an elevated vaginal pH without either an elevated Nugent score or the other portions of Amsel's criteria with poor outcomes including preterm birth and preterm premature rupture of membranes [19–21]. It is for this reason that we will be exploring our obstetrical outcomes by vaginal pH in this unique cohort.

## Figures and Tables

**Table 1:** Demographics.

Variable		95% CI
Age in years	23.7	22.7 to 24.7
Gravidity	2.5	1.9 to 3.1
Parity	1.4	1.3 to 1.5
Height in cm	151.2	150.6 to 151.8
Weight in kg	46.0	45.2 to 46.9
Years in school	8.8	8.5 to 9.2
Respiratory disease	1.57%	
Cardiac disease	0.94%	
Diabetes	0.31%	
Other diseases	0.63%	
